# Supplementary material for: Urban-rural inequalities and spatial arrangement of informed choice of family planning in Ethiopia: Further analysis of 2016 Ethiopian demographic health survey
Source: PLoS One. 2023 Aug 22;18(8):e0289099. doi: 10.1371/journal.pone.0289099 (PMC10443855; doi:10.1371/journal.pone.0289099)
Supplement: Attachment — Submitted filename: INF-choice.docx [file pone.0289099.s002.docx]

Dear editors.

Thank you very much for this opportunity to review this paper. This paper assessed urban-rural disparities and spatial distribution of informed choice of FP in Ethiopia, which is crucial for plociy makers and program managers to identify intervention on inequality.

The following are my suggestions for the improvement of this manuscript:

**General comments**

The authors don't include page numbers and lines in the document, making it difficult to give suggestions and comments by page and line

Make sure to leave space before each bracket or parenthesis

There is typo and spelling error thoughout the document

**Introduction.**

The introduction part must be rewritten orked and organised in a logical way. The aouthors must highlights what lloks the condition in Africa, then in Ethiopia. Incorporate national family planning strategy and initiatives, as well as health service accessibility and distribution by residence. Describe the efforts and gaps in providing access to quality family planning service to everyone who needs it. It should be discussed that how much govt has spent on family planning and benefit in terms of preventing unintended pregnancy and highrsik fertility behaviors

FP: to be defined first

**Method**

EDHS is used two stage stratified study design so there is a need for adjustment of cluster and weight while doing multivariate analysis. Need to elaborate the design of the study and whether any type of stratification has been carried out? In addition, the authors did a decomposition analysis but did not elaborate on it in the method section. Which residence coded as “0” or “1”? it need more details

**Independent variables**

Be consistent, for some variable athors mentioned the coatergy but form sone not yet

**Results**

Present the frequency of Table 1 by residence, then the total and weighted frequencies are enough.

**Decomposition analysis**

The authors stated that "the composition factors of region, religion, and place of residence were not included in the multivariable composition analysis because they had a p-value greater than 0.2 at the bivariable decomposition analysis". How could residence be an explanatory variable here? It was an urban-rural disparity study, hence the grouping variable in this case is residence.

In table one, I didn't notice a variable “place for the FP offer” to be classified as government or private, but in the decomposed table, I did?????

Correct the FF typoerror

“in the f informed choice”

“urbanand rural”

“by 9.3%.. Oppositely”

**Discusion**
It may be helpful to have a short summary of important findings in the first paragraph. The authors did not discuss limitations of the study such as the self-reported nature of the outcome informed choice of FP.

It is surprising that the composition of private health facilities had a narrowing effect, which indicates the composition of private health facilities was higher in rural areas, which is far from reality. Most private health facilities in Ethiopia are concentrated in urban areas.

**Conclusion**

State the the policy implications of this study based on the finding.
