# Supplementary material for: Urban-rural inequalities and spatial arrangement of informed choice of family planning in Ethiopia: Further analysis of 2016 Ethiopian demographic health survey
Source: PLoS One. 2023 Aug 22;18(8):e0289099. doi: 10.1371/journal.pone.0289099 (PMC10443855; doi:10.1371/journal.pone.0289099)
Supplement: Attachment — Submitted filename: point by point response.docx [file pone.0289099.s003.docx]

**Authors’ response to reviews**

# Title: Urban-rural inequalities and spatial arrangement of informed choice of family planning in Ethiopia: further analysis of 2016 Ethiopian demographic health survey.

Authors:

Abiyu Abadi Tareke (abiyu20010@gmail.com)

Bayley Adane Takele

_,_ Mohammedjud Hassen Ahmed

Masresha Derese Tegegne

Habitu Birhan Eshetu

Version: 1

Date: May 13, 2023

Point by point response for editors/reviewers’ comments

Manuscript number: PONE-D-23-01109

Dear editor/reviewer:

Dear all,

We would like to give deep gratitude for your constructive and fruitful comments. Your valuable comments enhanced content of the manuscript and we got highly experienced of scientific paper writing. The authors accounted each comment and clarification questions of editors and reviewers in a focused way. Our point-by-point responses to each comment and questions are detailed on the following pages. Further, the details of changes were shown under track changes in the supplementary document attached.

**Editors’ comment**

**Editor’s comment:** We note that Figure 3 in your submission contain map images which may be copyrighted.

**Authors’ answer:** Figure 3 in this manuscript was prepared using ArcGIS version 10.8 and SaTScan software by the authors of this manuscript. Shapefile (the **geospatial vector data format for geographic information system (GIS) software) were gotten from** <https://data.humdata.org/dataset/cod-ab-eth> website. This figure is not copyrighted and was totally prepared by the authors using this two software. The primary investigator of this article has published similar maps that were not copyrighted.

**Review Comments to the Author**

Using 2016 EDHS data the authors assessed urban-rural inequalities and spatial arrangement of informed choice of family planning in Ethiopia. The paper reported that spatial configuration of not informed choice was clustered and magnitude of informed choice was 12 percent higher in urban residents compared to rural residents.

**Reviewer 1**

**Title:**

**Reviewer’s comment:** It is not recommended to use abbreviation on the title: so better you write full form.

**Authors’ response: Dear reviewer, Thank you, for your informative comment here. We expand the abbreviation FP to family planning accordingly.**

**Abstract:**

**Reviewer’s comment:** correct editorial error such as not capitalizing the first letter of new sentence and capitalizing the middle word ‘’Descriptive’’. The same editorial issues are there under discussion.

**Authors’ response: Thank you for your constructive comment. We figure out the words and sentences having editorial and typography error and corrected it according to your comment.**

**Reviewer’s comment:** This sentence is incomplete and what authors want to say is not clear -More specifically, place of FP offers i.e., private health facilities (-16%), listening to the radio programs (+12%), age 35 to 49 years (-9.3%) and 25 to 34 years (+9.2%)	(p<0.05.

**Authors’ response: This incomplete sentence also confused us when we read it after your comments. We can’t be here without your comments. We rewrite it through subdividing it into two sentences as “Place of family planning offer i.e., private health facility, being aged between 35 and 49 years, and having visited to health facility in the last 1 year are found decreased the urban-rural gap of informed family planning choice by 15%, 9% and 5% respectively. Conversely, being aged between 25 and 34 years, being listener to radio have increased the gap by 9% and 12% respectively.”**

**Reviewer’s comment:** Check grammar for the following sentence:

In this study, visit private health facilities, women’s age group between 25 to 39 and visit to health facilities in the last 12 months factors that significantly widen urban - rural gap of family planning.

**Authors’ response: Thank you for your comment. we are sorry for confusing you. We tried to transfer the message “from this study variables such as having go to private health facilities, being woman age group between 25 to 39 and have been visited to health facilities in the last 12 months were variables that significantly widen the gap of informed family planning choices between urban and rural residents”. Dear reviewer, taking your comment as foundation to our correction, authors of this article agreed to change this sentence as “****the variables being private health facility visitor, being aged between 25 and 39 years and having visited health facilities in the last one year are found to increase the gap of informed family planning choices between urban and rural residents”**

**Reviewer’s comment:** You said: Besides, the spatial arrangement of not informed choice of family planning is not regular. To express spatial distribution the term random/non-random vs clustered are better rather than using not regular.

**Authors’ response: Dear reviewer we really appreciate your insight. To express spatial arrangement of not informed choice of family planning, words like non-random, random, evenly distributed and clustered are more expressive than not-regular. So, we decided to replace the word “not regular” by “not random”. See the corrected version of this manuscript.**

**Introduction:**

**Reviewer’s comment:** Check grammar issue in the following sentence:

- Informed choice of family planning means that when women choose a family planning method, all available information regarding side effects, what to in case of side effects, and possible alternative methods

**Authors’ response: Dear reviewer thanks for your fruitful comments. We rewrote the full sentence as “*Informed choice of family planning is defined as when a woman chooses a family planning method after receiving information about the possible side effects, what to in case of side effects, and possible alternative methods by healthcare providers*”**

**Reviewer’s comment:** Write full form followed by abbreviations in bracket first time abbreviations appear in manuscript

**Authors’ response: We corrected it accordingly. See the revised version of the manuscript.**

**Reviewer’s comment:** Check grammar issue in the following sentence:

- Moreover, the current study identified the spatial distribution and identified hotspots areas where women who had uninformed family planning choices.

**Authors’ response: Dear reviewer we rewrite it the sentence to *“In addition to that, this study determined the spatial distribution of uninformed choice of family planning in Ethiopia and identified areas with high clustering of uninformed choice (hotspots) which are helpful in designing location specific interventions.”***

**Methods:**

**Reviewer’s comment:** Even if the authors used secondary data some sections of methods were absent. Please add subsections on study area, design, population and data source

**Authors’ response: Thank you for your constructive comments. We included study area, study period, population, data source in the new version of the manuscript after your comment.**

**Reviewer’s comment:** Editorial issue: don’t capitalize letters in the middle of the sentence “Visit”

**Authors’ response: We given correction accordingly. See the revised version of the manuscript.**

**Results:**

**Reviewer’s comment:** In figure 1 title mention time component, you can use Year EDHS was released. Similarly for table 1 title is incomplete address where and when component.

**Authors’ response: Thank you. We modified the figure 1title as “Distribution of informed family planning choices by place of residence in Ethiopia, 2016.” And the title of Table 1 as “socio-demographic characteristics of study participants in Ethiopia, 2016.”**

**Reviewer’s comment:** Under the following section the authors wrongly cited Figure 1 replace it with Figure 2 because there are no p value in Figure 1 “**The spatial arrangement of the uninformed choice of FP”**

**Reviewer’s comment:** Similarly in the paragraph under figure 2, omit figure 2 (wrongly cited)

**Authors’ response: Dear reviewer this comment is huge input to our article. Thank you for your decisive comments. We solved and corrected the wrongly cited figures accordingly. The revised version of this manuscript.**

**Discussion**

**Reviewer’s comment:** Check grammar issue in the following sentence:

In this study, being in the age group 35 to 49 years was significantly narrowed the urban and rural gap in the informed choice of family planning where as being in the age group 25 to 34 found to widen the urban -rural gap of family planning compared to age group 15 to 24 year.

**Authors’ response: thank you reviewer for your comments. We appreciate your valuable comment. We corrected the sentence as “*Compared to age group of 25 to 34 years being age group between 35 and 49 years is found decreasing the gap of informed choice of family planning between rural and urban residents***.**”**

**Reviewer’s comment:** Try to include strength and limitations of this study

**Authors’ response: we acknowledged important strengths and limitations of this study to the new version of this manuscript.**

**Conclusions**

**Reviewer’s comment:** The first sentence is unnecessary

**Authors’ response: Dear reviewer, after your suggestion, we understood that the first sentence of the conclusion part of this article talks about methods and sample size of the article and this sentence is not based on the finding of the article. We agreed to remove it.**

**Declarations**

**Reviewer’s comment:** You specified only single abbreviations but there are a lot of abbreviations inside document. So, include all abbreviations-SNNP, NGO, FP……

**Authors’ response: Failed to list all abbreviations was our huge miss of the article. Dear reviewer we appreciate your comment. In the new revised version of this manuscript, we included all the utilized abbreviations in declaration section.**

**Reviewer 2**

Dear editors.

Thank you very much for this opportunity to review this paper. This paper assessed urban-rural disparities and spatial distribution of informed choice of FP in Ethiopia, which is crucial for plociy makers and program managers to identify intervention on inequality.

The following are my suggestions for the improvement of this manuscript:

**General comments**

The authors don't include page numbers and lines in the document, making it difficult to give suggestions and comments by page and line

Make sure to leave space before each bracket or parenthesis

There is typo and spelling error thoughout the document

**Introduction.**

The introduction part must be rewritten orked and organised in a logical way. The aouthors must highlights what lloks the condition in Africa, then in Ethiopia. Incorporate national family planning strategy and initiatives, as well as health service accessibility and distribution by residence. Describe the efforts and gaps in providing access to quality family planning service to everyone who needs it. It should be discussed that how much govt has spent on family planning and benefit in terms of preventing unintended pregnancy and highrsik fertility behaviors

FP: to be defined first

**Authors’ response:** dear reviewer we re-organize the introduction part according to your suggestion and fruitful comments.

**Method**

EDHS is used two stage stratified study design so there is a need for adjustment of cluster and weight while doing multivariate analysis. Need to elaborate the design of the study and whether any type of stratification has been carried out? In addition, the authors did a decomposition analysis but did not elaborate on it in the method section. Which residence coded as “0” or “1”? it needs more details

**Authors’ response: this comment is favorable additive to our article. We added details of study design, procedure of analysis and weighting measures. See the new revised version of manuscript.**

**Independent variables**

Be consistent, for some variable authors mentioned the coatergy but form sone not yet

**Results**

Present the frequency of Table 1 by residence, then the total and weighted frequencies are enough.

**Decomposition analysis**

The authors stated that "the composition factors of region, religion, and place of residence were not included in the multivariable composition analysis because they had a p-value greater than 0.2 at the bivariable decomposition analysis". How could residence be an explanatory variable here? It was an urban-rural disparity study, hence the grouping variable in this case is residence.

**Authors’ response: dear reviewer we are sorry about this confusion. We have been utilizing the word region and residence interchangeably mistakenly. Here residence means region. But, those two variables are not the same. We removed the variable “residence” from this sentence. Residence is not our independent variable but variable to be explained.**

In table one, I did not notice a variable “place for the FP offer” to be classified as government or private, but in the decomposed table, I did?????

**Authors’ response: thank you for your comment. We putted the variable place of FP offer as “Source of family planning” in the socio demographic table. In the new revised version of the manuscript, we rewrite it as “place of FP offer.” Sorry for the inconsistent use of variable name.**

Correct the FF typoerror

“in the f informed choice”

“urbanand rural”

“by 9.3%. Oppositely”

**Reviewers’ response: dear reviewer we checked all your comments and corrected accordingly.**

**Discussion**
It may be helpful to have a short summary of important findings in the first paragraph. The authors did not discuss limitations of the study such as the self-reported nature of the outcome informed choice of FP.

**Authors’ response: dear reviewer after getting your suggestion to include limitations, we included all possible limitations and bias that might occurred in this manuscript. We also added some important findings summary to the frist paragraph of the discussion session.**

It is surprising that the composition of private health facilities had a narrowing effect, which indicates the composition of private health facilities was higher in rural areas, which is far from reality. Most private health facilities in Ethiopia are concentrated in urban areas.

**Authors’ response: thank you reviewer for your concern. This finding might be pradox for readers. But receiving any health services at private health facilities is good informed choice of services. This is because of at private HF patient follow is not that much as crowed as public health facilities. If the patient follow rate is low then the care provider has opportunity to offer knowledge based well informed services to their clients. In addition, to this as private HF is profitable organization, care providers are eagar to offer informed choice to their client to attract more clients.**

**Conclusion**

State the the policy implications of this study based on the finding.

**Authors’ response: thank you for your comment. we corrected accordingly.**
