# Supplementary material for: Urban-rural inequalities and spatial arrangement of informed choice of family planning in Ethiopia: Further analysis of 2016 Ethiopian demographic health survey
Source: PLoS One. 2023 Aug 22;18(8):e0289099. doi: 10.1371/journal.pone.0289099 (PMC10443855; doi:10.1371/journal.pone.0289099)
Supplement: Attachment — Submitted filename: Response to Reviewers.docx [file pone.0289099.s004.docx]

**Authors’ response to reviews**

# Title: Urban-rural inequalities and spatial arrangement of informed choice of family planning in Ethiopia: further analysis of 2016 Ethiopian demographic health survey.

Authors:

Abiyu Abadi Tareke (abiyu20010@gmail.com)

Bayley Adane Takele

_,_ Mohammedjud Hassen Ahmed

Masresha Derese Tegegne

Habitu Birhan Eshetu

Version: 2

Date: May 13, 2023

Point by point response for editors/reviewers’ comments

Manuscript number: PONE-D-23-01109

Dear editor/reviewer:

Dear all,

We express our profound appreciation for the insightful and productive feedback that you have provided. Your invaluable comments have significantly enriched the quality of the manuscript, and have greatly augmented our expertise in the realm of scientific paper writing. The authors have diligently taken into account each of the comments and queries raised by the editors and reviewers, and have responded to them in a targeted manner. Our comprehensive point-by-point rejoinders to all the comments and questions can be found in the subsequent pages. In addition, an accompanying supplementary document has been enclosed, which showcases the modifications made in detail, using the track changes feature. We also made some change to fix grammatical error in some paragraphs.

**Review Comments to the Author**

**Reviewer’s comment:** see carefully the age group in the abstract section particularly at result and conclusion section (35 to 49 VS 25 to 39).

**Authors’ response:** dear reviewer we are here because of your insight and deep review to this manuscript. Sorry for this typographical error. After checking the result written in the regression table, we changed the phrase **“being aged between 25 and 39 years”** to **“being aged between 35 and 49 years”.**
