# Supplementary material for: Urban-rural inequalities and spatial arrangement of informed choice of family planning in Ethiopia: Further analysis of 2016 Ethiopian demographic health survey
Source: PLoS One. 2023 Aug 22;18(8):e0289099. doi: 10.1371/journal.pone.0289099 (PMC10443855; doi:10.1371/journal.pone.0289099)
Supplement: Attachment — Submitted filename: REVIEWER COMMENT.docx [file pone.0289099.s001.docx]

Review Comments to the Author

Using 2016 EDHS data the authors assessed urban-rural inequalities and spatial arrangement of informed choice of family planning in Ethiopia. The paper reported that spatial configuration of not informed choice was clustered and magnitude of informed choice was 12 percent higher in urban residents compared to rural residents.

**Title:**

It is not recommended to use abbreviation on the title: so better you write full form.

Abstract: correct editorial error such as not capitalizing the first letter of new sentence and capitalizing the middle word ‘’Descriptive’’. The same editorial issues are there under discussion.

This sentence is incomplete and what authors want to say is not clear -More specifically, place of FP offers i.e., private health facilities (-16%), listening to the radio programs (+12%), age 35 to 49 years (-9.3%) and 25 to 34 years (+9.2%)	(p<0.05.

Check grammar for the following sentence:

In this study, visit private health facilities, women’s age group between 25 to 39 and visit to health facilities in the last 12 months factors that significantly widen urban - rural gap of family planning.

You said: Besides, the spatial arrangement of not informed choice of family planning is not regular. To express spatial distribution the term random/non-random vs clustered are better rather than using not regular.

**Introduction:**

Check grammar issue in the following sentence:

- Informed choice of family planning means that when women choose a family planning methods, all available information regarding side effects, what to in case of side effects, and possible alternative methods
- Write full form followed by abbreviations in bracket first time abbreviations appear in manuscript

Check grammar issue in the following sentence:

- Moreover, the current study identified the spatial distribution and identified hotspots areas where women who had uninformed family planning choices.

**Methods:**

- Even if the authors used secondary data some sections of methods were absent. Please add subsections on study area, design, population and data source
- Editorial issue: don’t capitalize letters in the middle of the sentence “Visit”

**Results:**

- In figure 1 title mention time component, you can use Year EDHS was released. Similarly for table 1 title is incomplete address where and when component.
- Under the following section the authors wrongly cited Figure 1 replace it with Figure 2 because there are no p value in Figure 1 “**The spatial arrangement of the uninformed choice of FP”**
- Similarly in the paragraph under figure 2, omit figure 2 (wrongly cited)

**Discussion**

- Check grammar issue in the following sentence:

In this study, being in the age group 35 to 49 years was significantly narrowed the urban and rural gap in the informed choice of family planning where as being in the age group 25 to 34 found to widen the urban -rural gap of family planning compared to age group 15 to 24 year.

- Try to include strength and limitations of this study

**Conclusions**

- The first sentence is unnecessary

**Declarations**

You specified only single abbreviations but there are a lot of abbreviations inside document. So include all abbreviations-SNNP, NGO, FP……

References

Some references are too old (16=1996, 24=2007, 28=2001) . Try to use recent publications
